# Outcomes of Lower Limb Free Flap Reconstruction in a UK Major Trauma Centre: Acute Trauma, Delayed Breakdown, and Late Elective Reconstruction for Infected Metalwork

**DOI:** 10.7759/cureus.105670

**Published:** 2026-03-22

**Authors:** Pouya Mafi, Alicja Moore, Conor Taylor, Shirrushtii Appan, Sarvnaz Sepehripour, Mohamed Maher

**Affiliations:** 1 Plastic Surgery, Queen Elizabeth Hospital Birmingham, Birmingham, GBR

**Keywords:** acute lower limb trauma, arterial anastomosis, free flap reconstruction, infected metalwork, lower limb reconstruction, microsurgery, orthoplastic surgery, return to theatre, soft tissue reconstruction

## Abstract

Background

Free flap reconstruction is integral to the management of complex lower limb trauma, yet flap failure and return to theatre remain clinically significant challenges. Outcomes may vary according to injury timing and context.

Methods

A retrospective cohort study was conducted at a UK major trauma centre, including patients who underwent lower limb free flap reconstruction over a five-year period. Patients were categorised into three predefined groups: acute trauma reconstruction, closed fractures with delayed soft-tissue breakdown, and late elective reconstruction for infected metalwork. The primary outcome for acute trauma was any flap loss. A composite reconstruction outcome was used for the infected metalwork cohort. Descriptive and univariable statistical analyses were performed.

Results

Seventy patients underwent acute lower limb free flap reconstruction. Any flap loss occurred in five cases (7.1%), including four total and one partial flap loss. Return to theatre occurred in 31.4% of cases, with all flap losses occurring in patients requiring re-exploration. No flap losses were observed in patients with initially closed fractures who later developed soft-tissue breakdown (0/10). Nineteen patients underwent late elective free flap reconstruction for infected metalwork; partial or total flap loss occurred in 31.6%, and 57.9% achieved a satisfactory reconstruction using a predefined composite outcome.

Conclusions

Lower limb free flap reconstruction performed within a UK major trauma centre achieves flap survival rates comparable to published benchmarks despite managing complex injuries. Distinct biological contexts, including delayed breakdown and chronic infection, demonstrate differing outcome profiles, highlighting the importance of stratified analysis when evaluating reconstructive success.

## Introduction

Lower limb trauma remains a major cause of morbidity, often requiring complex reconstruction to restore limb integrity and function. The evolution of orthoplastic care in the United Kingdom has emphasised early, coordinated management of skeletal and soft-tissue injury, with free tissue transfer forming a cornerstone of reconstruction in severe cases. Despite advances in microsurgical technique and peri-operative care, free flap failure continues to represent a significant complication, with implications for limb salvage, patient outcomes, and healthcare resources [1,2].

Reported rates of free flap failure in lower limb reconstruction vary widely, reflecting differences in injury severity, timing, reconstructive strategy, and institutional experience [1,3,4]. Return to theatre (RTT) for flap compromise is common and represents both an opportunity for salvage and a marker of increased risk of failure [1]. Understanding the factors associated with flap survival and failure remains critical for optimising patient selection, operative planning, and postoperative management.

In addition to acute trauma reconstruction, a subset of patients present later with soft-tissue failure following initially closed fractures or internal fixation. These scenarios, including delayed wound breakdown and chronic infection associated with metalwork, represent distinct biological challenges compared with acute open injuries [5]. Outcomes in these settings are less well characterised, and extrapolation from acute trauma data may be inappropriate [5].

The aim of this study was to evaluate outcomes of lower limb free flap reconstruction at a UK major trauma centre and to explore whether clinical context-acute reconstruction versus delayed salvage for infected metalwork-affects reconstructive success, flap survival, and limb salvage. By analysing acute trauma reconstructions separately from delayed breakdown following closed fractures and late elective reconstruction for infected metalwork, this study seeks to provide a nuanced and clinically relevant assessment of free flap performance across different reconstructive contexts.

## Materials and methods

Study design and setting

This was a retrospective cohort study of patients undergoing lower limb free flap reconstruction at Queen Elizabeth Hospital Birmingham, a UK tertiary major trauma centre, over a five-year period. The centre receives some of the most complex lower limb trauma referrals nationally and delivers consultant-led orthoplastic care, with senior orthopaedic and plastic surgery consultants jointly involved in decision-making and operative management in accordance with UK standards for the management of open lower limb fractures. Comprehensive facilities were available across the entire patient pathway, including dedicated trauma theatres, inpatient services, outpatient clinics, and structured follow-up.

Case identification and data source

Eligible cases were identified using the Oceano electronic system to generate a list of hospital numbers corresponding to patients who underwent free flap reconstruction in the given period. Individual medical and operative records and trauma databases were reviewed in detail to verify the above. Data collected included demographic characteristics, injury characteristics, timing of debridement and flap coverage, flap type, operative variables, complications, RTT, flap loss, and limb salvage outcomes. Postoperative outcomes were assessed through review of electronic patient records and outpatient follow-up documentation.

Eligibility criteria

Patients were included if they had undergone free flap reconstruction for lower limb trauma within the preceding five years and had complete clinical and operative documentation suitable for predefined variable extraction. Patients were excluded if reconstruction did not involve the lower limb, if documentation was incomplete or inconsistently recorded, if open lower limb fractures were managed with debridement and direct primary closure without free flap reconstruction, or if soft-tissue reconstruction was performed using local flaps, biodegradable temporising matrix (BTM), split-thickness skin grafts, or non-operative management rather than free tissue transfer. These exclusions were applied to ensure a homogeneous cohort restricted to injuries requiring free flap reconstruction.

Cohort definitions

To preserve biological and temporal homogeneity, patients were categorised into three predefined groups. The primary cohort consisted of patients undergoing acute free flap reconstruction as part of initial orthoplastic management for lower limb trauma. A further predefined subgroup comprised of patients with initially closed fractures who subsequently developed delayed soft-tissue breakdown requiring free flap reconstruction. And finally, the secondary cohort included patients undergoing delayed free flap reconstruction weeks to years following fracture fixation, without an initial open wound, for complications such as metalwork failure, chronic deep infection, sinus formation, or wound breakdown. The secondary cohort was analysed separately and descriptively because of its distinct biological profile and delayed reconstructive timing.

Variables and definitions

Demographic variables included age and sex. Pre-operative risk factors included individual comorbidities as documented in clinical records, including type 2 diabetes mellitus, hypertension, ischaemic heart disease, atrial fibrillation, heart failure, chronic obstructive pulmonary disease, multiple sclerosis, and benign prostatic hyperplasia. Injury severity was categorised as high-energy or low-energy based on the mechanism of injury. Operative variables included flap type, recipient vessel, arterial anastomosis configuration (end-to-end versus end-to-side), number of venous anastomoses where documented, and ischaemia time when recorded. For the acute trauma cohort, timing variables included time from injury to first debridement and time from injury to definitive free flap reconstruction.

Outcomes

For the acute trauma cohort, the primary outcome was any flap loss, defined as total flap failure or partial flap necrosis. Partial flap losses were further described according to the need for additional reconstructive intervention. For the secondary infected metalwork cohort, a composite reconstruction outcome was used. Reconstruction was classified as satisfactory only when explicitly documented as such in the outcome records. All other outcomes-including major amputation, requirement for skin grafting or dermal substitutes, chronic wounds, or persistent/recurrent infection-were classified as failed reconstruction. RTT was recorded as a postoperative course variable reflecting flap compromise and salvage attempts.

Statistical analysis

Continuous variables were summarised as mean ± standard deviation and median with interquartile range (IQR), and compared using non-parametric methods (Mann-Whitney U test) where appropriate. Categorical variables were summarised as frequencies and percentages and compared using Fisher’s exact test. Univariable logistic regression analysis was performed to estimate odds ratios (ORs) with 95% confidence intervals (CIs) for continuous predictors of flap loss. Where zero cells occurred in contingency tables, continuity correction was applied. Owing to the limited number of flap loss events, multivariable modelling was not performed. A two-tailed p-value < 0.05 was considered statistically significant. The secondary infected metalwork cohort was analysed descriptively without inferential comparison to the acute trauma cohort.

## Results

Study cohorts

A total of 89 patients were identified across all datasets. Following application of predefined inclusion and exclusion criteria, three biologically distinct cohorts were analysed. The primary cohort consisted of 70 patients who underwent acute lower limb free flap reconstruction following trauma. Within this group, 10 patients sustained initially closed fractures that subsequently developed soft-tissue breakdown requiring free flap reconstruction and were analysed as a predefined subgroup. A secondary cohort comprised 19 patients who underwent late elective free flap reconstruction for infected metalwork, deep infection, sinus formation, or wound breakdown following internal fixation of lower limb fractures. Two cases involving local flap reconstruction were excluded from the primary cohort. One case in the infected metalwork cohort was excluded due to incomplete outcome data. All reconstructions analysed represent free tissue transfer procedures.

Primary cohort: acute lower limb free flap reconstruction (N = 70)

The acute trauma cohort consisted of 70 patients with a mean age of 49.7 ± 20.2 years and a median age of 52.5 years (IQR 32.0-68.5). Fifty patients (71.4%) were male, and 20 (28.6%) were female. The median time from injury to first surgical debridement was 1.0 day (IQR 0-2), and the median time from injury to definitive free flap reconstruction was 5.5 days (IQR 4-9). Ischaemia time was recorded in 62 cases (88.6%), with a median duration of 62.5 minutes (IQR 47.25-80.0). Baseline demographic and peri-operative characteristics are summarised in Table 1.

**Table 1:** Baseline demographic and peri-operative characteristics Descriptive statistics for the acute trauma free flap group. Continuous variables are presented as mean ± SD and median (IQR) IQR: interquartile range; SD: standard deviation

Variable	Value
Age (years), mean ± SD	49.7 ± 20.2
Male sex, n (%)	50 (71.4%)
Time to first debridement (days), median (IQR)	1.0 (0-2)
Time to flap (days), median (IQR)	5.5 (4-9)
Ischaemia time recorded, n (%)	62 (88.6%)
Ischaemia time (min), median (IQR)	62.5 (47.25-80.0)

Any flap loss, defined as total or partial flap necrosis, occurred in five of 70 cases (7.1%). Total flap loss occurred in four cases (5.7%), and partial flap loss occurred in one case (1.4%). The anterolateral thigh (ALT) flap was the most frequently performed reconstruction, accounting for 48 cases (68.6%). Radial forearm free flaps were performed in 10 cases (14.3%), gracilis muscle flaps in six cases (8.6%), and serratus anterior flaps in two cases (2.9%). Single cases of medial sural artery perforator and parascapular flaps were also recorded. All five flap losses occurred in ALT flaps (5/48, 10.4%), with no flap losses observed amongst other flap types (Table 2).

**Table 2:** Details of flap type distribution and outcomes Flap type and flap loss rates in the acute trauma free flap cohort. ‘Any flap loss’ includes total flap loss and partial flap necrosis ALT: anterolateral thigh; RFFF: radial forearm free flap; MSAP: medial sural artery perforator

Flap type	n (%)	Any flap loss n (%)
ALT	48 (68.6%)	5 (10.4%)
RFFF	10 (14.3%)	0
Gracilis	6 (8.6%)	0
Serratus	2 (2.9%)	0
MSAP	1 (1.4%)	0
Parascapular	1 (1.4%)	0
Other free flap	2 (2.9%)	0

Injury mechanism was classified as high-energy or low-energy. High-energy injuries accounted for 30 cases, with four flap losses (13.3%), whilst low-energy injuries accounted for 40 cases, with one flap loss (2.5%). Fisher’s exact test (two-tailed) demonstrated no statistically significant association between injury energy and flap loss (p = 0.157). Arterial anastomosis configuration was end-to-end in 28 cases and end-to-side in 42 cases. Flap loss occurred in one case following end-to-end anastomosis (3.6%) and in four cases following end-to-side anastomosis (9.5%). Fisher’s exact testing demonstrated no statistically significant association between anastomotic configuration and flap loss (p = 0.641). RTT occurred in 22 of 70 cases (31.4%). All five flap losses occurred in patients who required RTT (5/22, 22.7%), whereas no flap losses occurred amongst patients who did not require RTT (0/48, 0%). Fisher’s exact test demonstrated a statistically significant association between RTT and flap loss (p = 0.002). These results are summarised in Table 3.

**Table 3:** Categorical predictors of flap loss in the primary cohort

Variable	Any flap loss n/N (%)	p-value
High-energy injury	4/30 (13.3%)	0.157
Low-energy injury	1/40 (2.5%)	
End-to-side anastomosis	4/42 (9.5%)	0.641
End-to-end anastomosis	1/28 (3.6%)	
Return to theatre	5/22 (22.7%)	0.002
No return to theatre	0/48 (0%)	

The posterior tibial artery was the most commonly used recipient vessel (55 cases), with four flap losses (7.3%). The anterior tibial artery was used in 10 cases with no flap losses. Other recipient vessels accounted for five cases, with one flap loss. The number of venous anastomoses was recorded in 54 cases. One venous anastomosis was performed in 32 cases, amongst which four flap losses occurred (12.5%), whilst two venous anastomoses were performed in 22 cases, with one flap loss (4.5%).

Continuous variables were compared between cases with and without flap loss using the Mann-Whitney U test. There was no difference in median time to first debridement between groups (U = 191.0, p = 0.501). Median time to definitive flap reconstruction was 4.0 days (IQR 4-9) in cases with flap loss and 6.0 days (IQR 4-9) in cases without flap loss (U = 160.5, p = 0.973). Ischaemia time analysis included only cases with recorded values (n = 62); median ischaemia time was 47.0 minutes (IQR 30-57) in cases with flap loss and 64.0 minutes (IQR 50-80) in cases without flap loss (U = 73.0, p = 0.074). Continuous variable analyses are presented in Table 4.

**Table 4:** Continuous predictors of flap loss in the primary cohort Continuous predictors were compared between the flap loss and flap survival groups using the Mann-Whitney U test. U and Z statistics are shown with two-tailed p-values. Statistical significance was defined as p < 0.05 IQR: interquartile range

Variable	Flap loss median (IQR)	No loss median (IQR)	Mann-Whitney U	Z	p-value
Time to first debridement (days)	1.0 (1-2)	1.0 (0-2)	191.0	0.650	0.501
Time to flap (days)	4.0 (4-9)	6.0 (4-9)	160.5	−0.046	0.973
Ischaemia time (min)	47.0 (30-57)	64.0 (50-80)	73.0	−1.797	0.074

Individual comorbidities were analysed separately. Type 2 diabetes mellitus was present in seven patients, amongst whom one flap loss occurred (14.3%). Hypertension was present in 15 patients, with two flap losses (13.3%). Other recorded comorbidities, including ischaemic heart disease, atrial fibrillation, heart failure, chronic obstructive pulmonary disease, multiple sclerosis, and benign prostatic hyperplasia, were infrequent, and no flap losses were observed in these subgroups. Comorbidity-specific outcomes are shown in Table 5.

**Table 5:** Comorbidities and flap outcomes in the primary cohort

Comorbidity	n	Any flap loss n (%)
Type 2 diabetes mellitus	7	1 (14.3%)
Hypertension	15	2 (13.3%)
Other comorbidities	≤3 each	0

Within the primary cohort, 10 patients sustained initially closed fractures that subsequently developed soft-tissue breakdown requiring free flap reconstruction. No flap losses occurred in this subgroup (0/10, 0%). In the remaining acute trauma cohort (n = 60), five flap losses (8.3%) were observed. This comparison is presented in Table 6.

**Table 6:** Closed fracture with delayed breakdown subgroup

Group	N	Any flap loss n (%)
Closed → breakdown	10	0 (0%)
Remaining acute cohort	60	5 (8.3%)

Secondary cohort: late elective infected metalwork reconstruction (N = 19)

Nineteen patients underwent late elective free flap reconstruction for metalwork failure, deep infection, sinus formation, or wound breakdown following internal fixation of lower limb fractures. The mean age was 47.2 ± 14.6 years, and 14 patients (73.7%) were male. Ischaemia time was recorded in 14 cases (73.7%), with a median duration of 67.5 minutes (IQR 46.5-78.0). RTT occurred in nine cases (47.4%).

Any flap loss occurred in six of 19 cases (31.6%). Using the predefined composite reconstruction outcome, 11 patients (57.9%) achieved satisfactory reconstruction, whilst eight patients (42.1%) were classified as having failed reconstruction. Major amputation was required in five cases (26.3%). Secondary cohort outcomes are summarised in Table 7.

**Table 7:** Secondary cohort outcomes: infected metalwork reconstructions

Outcome	n (%)
Satisfactory reconstruction	11 (57.9%)
Composite reconstruction failure	8 (42.1%)
Any flap loss	6 (31.6%)
Major amputation	5 (26.3%)
Return to theatre	9 (47.4%)

## Discussion

This study presents outcomes of lower limb free flap reconstruction from a UK major trauma centre, with analysis structured around biologically distinct cohorts: acute trauma reconstruction, delayed breakdown following initially closed fractures, and late elective reconstruction for infected metalwork. By separating these entities, the study provides a clinically realistic appraisal of flap performance across different reconstructive contexts whilst avoiding inappropriate pooling of heterogeneous pathologies.

Acute lower limb free flap reconstruction in a UK major trauma centre

In the primary cohort, the rate of any flap loss was 7.1%, with a total flap loss rate of 5.7%. These outcomes compare favourably with contemporary published series and systematic reviews reporting flap failure rates in lower limb reconstruction generally ranging between 5% and 10% [1]. A recent systematic review by Serra et al. reported pooled flap failure rates and highlighted substantial variability across centres, with RTT rates frequently exceeding 25% [1]. Within this context, the results of the present cohort fall well within expected international benchmarks whilst reflecting the complexity typical of a tertiary UK major trauma centre.

The RTT rate of 31.4% observed in this cohort is consistent with the burden of high-energy trauma and extensive soft-tissue injury managed at specialist centres. Importantly, all flap losses occurred in patients requiring RTT, whereas no flap losses were observed amongst patients who did not undergo re-exploration. This pattern reflects established clinical practice whereby RTT is not a predictor in isolation but a marker of early vascular compromise prompting salvage attempts. Prior studies, including those summarised by Serra et al. [1] and Struebing et al. [6], demonstrate that early re-exploration remains central to flap salvage strategies, with successful outcomes achievable in a substantial proportion of cases.

Anastomotic configuration, recipient vessels, and technical factors

No statistically significant association was identified between arterial anastomotic configuration (end-to-end versus end-to-side) and flap loss. This finding is consistent with existing literature demonstrating comparable flap survival between these techniques when used appropriately [1,6]. The choice of anastomosis in lower limb trauma is often dictated by vessel quality, distal perfusion requirements, and zone of injury rather than a single preferred configuration, and the present data support the safety of both approaches within a consultant-led orthoplastic setting.

Similarly, recipient vessel selection was not associated with differential flap survival. The posterior tibial artery was the most commonly used recipient vessel, reflecting standard reconstructive practice in the lower limb. The absence of flap loss in anterior tibial artery anastomoses should be interpreted cautiously, given the small number of cases and selection bias inherent to vessel choice.

All flap losses occurred in ALT flaps, which represented the majority of reconstructions. This likely reflects flap utilisation rather than flap-specific risk. Larger flap size has been associated with increased complication rates in lower limb reconstruction, as demonstrated by Lee et al. [7], and may partially explain this distribution without implying inferiority of the ALT flap itself. The ALT flap remains a versatile workhorse for lower limb reconstruction, particularly in complex traumatic defects requiring substantial tissue volume.

Timing, ischaemia, and physiological considerations

No association was identified between flap loss and time to first debridement or time to definitive flap reconstruction within the acute cohort. These findings should be interpreted in the context of the UK lower limb open fracture management standards, where early debridement and timely soft-tissue coverage are prioritised, limiting variability in timing across patients. The narrow distribution of timing variables likely reduces the ability to detect differences within this cohort.

Ischaemia time was shorter in cases with flap loss compared with surviving flaps, although this difference did not reach statistical significance. This counterintuitive finding underscores the limitation of interpreting isolated intra-operative metrics in small cohorts and highlights the multifactorial nature of flap failure. As described by Yoon and Jones [8], flap survival following vascular compromise depends not only on ischaemia duration but also on neovascularisation and timing of compromise relative to angiogenic maturation. These physiological processes may influence outcomes beyond what is captured by raw ischaemia time alone.

Closed fractures with delayed soft-tissue breakdown

No flap losses occurred in the subgroup of patients with initially closed fractures that later developed soft-tissue breakdown requiring free flap reconstruction. Whilst underpowered for definitive inference, this finding suggests that delayed breakdown following closed injury does not necessarily confer increased risk of flap loss when managed within a specialist reconstructive pathway. These cases likely represent a distinct biological process compared with acute open trauma, characterised by secondary soft-tissue compromise rather than primary devitalisation and contamination.

Late elective reconstruction for infected metalwork

The secondary cohort of patients undergoing late elective free flap reconstruction for infected metalwork demonstrated markedly different outcomes. In this group, 11 patients (57.9%) achieved satisfactory reconstruction, whereas eight (42.1%) experienced composite reconstruction failure. Out of the 19 patients, nine patients (47.4%) returned to theatre. In total, there were six patients with partial or total flap loss (31.6%), and major amputation was required in five (26.3%). These outcomes reflect the hostile biological environment associated with chronic infection, multiple prior operations, compromised vascularity, and prolonged inflammatory burden.

Importantly, despite these challenges, satisfactory reconstruction was achieved in over half of the patients. This finding supports the role of microsurgical reconstruction even in late, complex salvage scenarios. The observed outcomes align with published systematic reviews by Koster et al. [9] and long-term institutional experience reported by Struebing et al. [6], which emphasise that flap failure and secondary amputation represent recognised endpoints within a staged reconstructive pathway rather than isolated technical failures.

Limitations

The limitations of this study include its retrospective design, relatively small cohort size, and heterogeneity of reconstructive indications. Data were dependent on the accuracy and completeness of clinical documentation, and cases with incomplete or non-reconcilable data were excluded, which may introduce selection bias. Although this approach ensured analytical reliability, it may have excluded patients with more complex or atypical clinical courses.

The primary acute trauma cohort contained a limited number of flap loss events, restricting statistical power and precluding multivariable regression analysis. As a result, associations identified in univariable analyses should be interpreted cautiously and cannot be assumed to represent independent predictors of flap failure. Similarly, subgroup analyses, including the closed fracture with delayed breakdown cohort, were underpowered and intended to be descriptive rather than inferential. Ischaemia time and venous anastomosis data were not recorded in all cases, limiting the completeness of these analyses. In addition, intra-operative variables such as flap size, perforator characteristics, and vessel quality were not consistently documented and therefore could not be analysed.

The secondary cohort of late elective free flap reconstruction for infected metalwork represents a biologically distinct population and was analysed descriptively. Comparisons between this cohort and the acute trauma cohort were not performed due to differences in timing, pathology, and outcome definitions. The composite outcome used for this cohort, whilst clinically meaningful, was based on documentation of reconstruction status and may not fully capture patient-reported outcomes or functional recovery.

Finally, this study reflects the experience of a single UK major trauma centre with established orthoplastic services and consultant-led care. Whilst this enhances internal validity, the findings may not be directly generalisable to centres with different trauma systems, case-mix, or reconstructive resources.

## Conclusions

Taken together, these findings illustrate that modern lower limb free flap reconstruction within a UK major trauma centre achieves outcomes comparable to international benchmarks despite managing some of the most complex injuries nationally. The separation of acute trauma, delayed breakdown, and chronic infection cohorts highlights the importance of biological context when interpreting reconstructive outcomes. Free flap failure remains uncommon in acute trauma when delivered within a coordinated orthoplastic service, whilst late elective reconstruction for infected metalwork represents a distinct reconstructive challenge with higher morbidity but meaningful salvage potential.
